# High Affinity Allele for the Gene of FCGR3A Is Risk Factor for HIV Infection and Progression

**DOI:** 10.1371/journal.pone.0015562

**Published:** 2010-12-20

**Authors:** Bhawna Poonia, Gustavo H. Kijak, C. David Pauza

**Affiliations:** 1 Division of Basic Science and Vaccine Research, Institute of Human Virology, Baltimore, Maryland, United States of America; 2 United States Military HIV Research Program/Henry M. Jackson Foundation, Rockville, Maryland, United States of America; University of Rochester, United States of America

## Abstract

**Background:**

We investigated the genetics of Fc receptors, which function as activating receptors on immune cells and help to control HIV through antibody-mediated cellular cytotoxicity. Thus, Fc receptors may be important for virus immunity but might also promote immune hyperactivation that would enhance infection.

**Methodology/Principal Findings:**

We measured abundance of low and high activity alleles in two Fc receptor genes, FCGR2A and FCGR3A, for persons with HIV disease, natural virus suppressors (HIV+, without disease) and healthy controls to show whether genotypes were associated with infection and disease. Individuals homozygous for the high activity allele of FCGR3A (158VV) were predominantly found among HIV progressors and this group was also skewed toward higher allele frequencies for the V158 variant. Both of the HIV positive groups (progressors and natural virus suppressors) had significantly higher frequencies of the V158 allele compared with uninfected controls. There were no apparent associations among FCGR2A alleles and HIV status.

**Conclusions/Significance:**

Our results indicate that high activity alleles of FCGR3A may be risk factors for HIV infection or progression and we need to understand how allelic variants affect the balance between virus control and immune activation.

## Introduction

The Fc receptors are a family of cell surface glycoproteins, which bind the constant regions (Fc) of soluble antibodies. They are implicated in diverse mechanisms of immune regulation. Cross-linking Fc receptors causes cell activation or inhibition depending on the individual receptor [1]. Fc receptors also help control the half life of circulating immunoglobulins by targeting bound antibodies to phagocytic or transport vesicles and participate in antibody-dependent cellular cytotoxicity (ADCC) or antibody-dependent cellular viral inhibition (ADCVI), a mechanism wherein FCR-dependent cell activation increases the production of chemokines that block HIV infection [2]. Fc receptors are also found on epithelial cells [3], [4], where they might be important for cell activation and mucosal immunity.

We are trying to understand the roles for Fc receptors in HIV transmission and disease, and how these functions would impact the potential for vaccines to protect against sexual transmission of this virus. Our approach is to explore the natural variation in Fc receptor genetics, testing for relationships between high or low activity alleles and HIV transmission or disease.

Two of the major receptors for IgG are the Fc γ receptor IIa (FCGR2A, CD32) and Fc γ receptor IIIa (FCGR3A, CD16). Allelic variants in both receptors are common and have been linked to multiple disease risks. A single nucleotide polymorphism (SNP) in FCGR3A, substituting histidine for arginine at position 131, was linked to recurring bacterial infection in children [5]. A valine for phenylalanine substitution at position 158 in FCGR3A was linked to the risk for autoimmune arthritis [6]. Both of these SNP affect antibody binding and signaling strength. Cancer patients treated with monoclonal antibodies Cetuximab or Rituximab had better responses if they carried high activity FcR alleles [7] ^,^ [8]. Studies on disease associations and responses to monoclonal antibody therapy demonstrate strong links between FCGR2A or FCGR3A genotype and clinical outcomes, and encourage studies on the roles for FCR in HIV transmission and disease.

We postulated that HIV transmission might be impacted by allelic variation in FCGR3A or FCGR2A, since these are important signaling receptors that will affect immune activation and susceptibility to infection. To test this hypothesis, we compared allele frequencies among groups with and without HIV infection. We also postulated that Fc receptor variation would affect disease progression. For this test, we compared a group of HIV-infected individuals with normally-progressing disease and a cohort of natural virus suppressors characterized by prolonged control of viremia without receiving antiretroviral therapy [9]. Our results showed surprising relationships between FCGR2A or FCGR3A genotypes and HIV disease.

## Results

The distribution of genotypes for FCGR3A SNP rs396991 among uninfected controls was similar to published reports for healthy African Americans [10] (42% FF, 50% VF and 8% VV) and Caucasian Americans [10] (50% FF, 39% VF and 11% VV) (Table 2). We noted a significant association between rs396991 and HIV status (Table 3). The presence of a V158 allele was significantly associated with HIV infection (p = 0.04 odds ratio 2.0 range 1.07–3.73). Uninfected controls had higher frequencies of FF homozygotes when compared with either of the HIV infected groups. Overall, the V158 allele was associated with an increased risk of HIV infection.

The VV genotype also seemed to be associated with HIV progression. The majority of VV genotype individuals (95%) were found among HIV progressors and the V158 allele frequency was highest in this group compared to uninfected or NVS groups. The VV genotype was rare among uninfected controls and was not found among natural virus suppressors. VF heterozygotes were more common among NVS or HIV progressors compared to uninfected controls.

The number of non-african American patients in our cohorts was small (3 in NVS and 5 in progressors). We analyzed the results by excluding them to have all African-american cohorts. Results again showed a higher frequency of V allele in HIV+ versus controls (p value 0.039, odds ratio 1.969 range 1.055−3.676).

It would be interesting to study if these FCGR genotypes influence correlates of HIV disease progression such as CD4 counts or viral loads, however, in our HAART treated cohorts, this analysis was not possible due to mostly undetectable viral RNA in plasma. The CD4 counts also reflect the impact of therapy and are not true measures of disease in these treated groups.

There was no association between FCGR2A rs1801274 genotypes and HIV sero-status. Among HIV sero-positive patients, variation in this locus was not associated with the NVS phenotype.

The FCGR2A FCGR3A extended haplotype showed no evidence of linkage disequilibrium among HIV sero-negative patients and the distribution of haplotypes was not significantly different among HIV-positive and HIV-negative patients. However, within the group of all HIV-positive patients, the RR∶FF double homozygous genotype was associated with HIV progression. Out of 60 HIV progressors, 11 had this combined genotype compared to 1/40 for NVS and 3/34 for HIV negative controls.

## Discussion

The results presented here showed that the V158 allele of FCGR3A was significantly associated with the risk for acquiring HIV infection. We did not observe an impact of FCGR2A alleles on HIV transmission in this admittedly small study. Thus, initial HIV transmission into virus naive, seronegative hosts, was more likely if the recipient carried high activity V158 alleles of FCGR3A.

A previous report showed that V158 was associated with the risk for Kaposi's sarcoma (KS) [11], a common indicator of HIV disease progression in the era before combination antiretroviral therapy. Since the advent of broadly available, combination antiretroviral therapy, HIV can be suppressed to low levels for prolonged intervals, the rates for KS have declined and it is now difficult to stratify HIV+ patients according to changing CD4 counts or vRNA levels. Accordingly, we elected to compare HIV+ individuals with progressing disease (requiring antiretroviral therapy) to our NVS group. We noted higher frequencies of FCGR3A V158 alleles among individuals with common disease. Our conclusions were similar to Lehrnbecher, et al., that V158 is associated with progressing HIV disease. Others showed that the low activity RR genotype in FCGR2A, was linked to CD4+ T cell counts declining below 200/mm^3^ [12], suggesting that the high activity alleles might be protective. Conversely, the high activity HH genotype was also related to the risk for *Pneumocystis* infection, which is an AIDS-defining illness and an independent marker of disease progression. Interestingly, perinatal HIV transmission may be affected by the infant's FcGR2A HH genotype [13]. The impact of FCGR2A variation on HIV acquisition and progression thus remains unclear.

Similarly, it is difficult to interpret the combined FCGR3A plus FCGR2A haplotype and its impact on HIV disease status. Although FCGR3A allele frequency was significantly associated with progressing disease compared to NVS donors, the combined haplotype of FCGR3A FF plus FCGR2A RR (both homozygous for low activity alleles) was significantly associated with the risk for progressing disease with an odds ratio of 8.76. This finding may indicate a strong requirement for FCGR2A (CD32) activity that is partially complemented by FCGR3A receptors. When individuals are homozygous with low activity alleles for both, capacity is decreased for HIV suppression that involves Fc receptors for IgG. However, when high activity alleles of FCGR3A are present, they also carry a risk for stronger immune activation that leads eventually to progressing disease.

Apparently, HIV transmission is enhanced among individuals with the V158 allele, even in the absence of virus-specific antibody. A previous study evaluated Fc receptor allelic variation and HIV infection among individuals who were vaccinated with HIV gp120 protein [14]. In that work, the FCGR2A H131 and FCGR3A V158 alleles were both related to increased risk for HIV infection. However, the amounts of IgG present in genital mucosa were likely to be low and may not have formed immune complexes with HIV. We do not support the view that virus-specific antibodies enhance HIV infection in persons carrying the V158 allele. We favor the interpretation that individuals with higher function FCGR3A will have increased immune activation and higher susceptibility to HIV transmission. This is similar to the conclusion in Lehrnbecher, et al, where they proposed that higher immune activation would promote KS [11]. Local immune activation might occur because of host antibody responses to another pathogen present in the mucosal epithelium or natural antibodies that are forming immune complexes with IgG and activating FCGR3A by cross-linking. The mechanism might involve cells that are not targets for HIV infection. For example, Lehner's group had postulated a role for Fc receptors on epithelial cells in the control of HIV transmission [3]. It was described recently that thymic stromal lymphopoietin (TSLP) production in epithelial cells was increased by HIV and could promote transmission to CD4+ target cells [15]. We are testing whether Fc receptor cross-linking will increase TSLP production by epithelium.

Although we are mostly interested in studying the effects of Fc receptor variation on immune activation and HIV disease, there are other possible mechanisms that are not ruled out. For example, antibody coating of HIV particles in the infected donor might increase the probability of virus transmission to a naïve host through interactions with FCR. Alternatively, some individuals may have natural antibodies that cross-react with HIV particles, similar to the commonly observed cross-reaction to HIV p24 [16]. Without additional studies on the mechanistic consequences of Fc receptor variation, these models cannot be eliminated.

Our studies can be improved by increasing the sample size and potentially, including other Fc receptor genes to create an expanded haplotype. We have not ruled out the possible impact on transmission or disease of genes that are close to the FCGR3A or FCGR2A loci. Further, we do not yet have experimental data from laboratory models that are relevant to the relationships of Fc receptor variants with HIV transmission or disease. Our ongoing studies are addressing these important issues.

## Materials and Methods

### Subjects

Blood samples for genomic DNA were obtained from 172 volunteers in three categories with respect to their HIV status (Table 1). Forty three specimens came from a Natural Virus Suppressor (NVS) cohort at the Institute of Human Virology [9]. NVS are HIV positive individuals who control HIV replication without antiretroviral therapy; they were confirmed HIV-positive by serology, detection of proviral DNA and for some cases, virus recovery from *in vitro* cultures but vRNA remains mostly below detection [9]. All members of this cohort are African American, reflecting the population of HIV-infected individuals at our clinics in Baltimore, MD. Another group of 59 patients with HIV disease were receiving antiretroviral therapy and are classified as progressors. These individuals report a variety of risk factors for HIV and likely include cases of sexual and intravenous transmission. We have classified them as progressors in order to distinguish them from the other HIV+ group of natural virus suppressors. The average times of infection for the NVS and progressor groups were 8.1 and 8.7 years, respectively. Another group included 70 HIV negative individuals, which served as uninfected controls.

**Table 1:** Study populations demographic.

	Controls (n = 70)	HIV positive	
		Progressors (n = 59)	NVS (n = 43)
Ethnicity	AA(93%)	AA(95%)	AA(100%)
Gender	M(38%), F(62%)	M(70%), F(30%)	M(49%), F(51%)
HIV status	Negative	Positive	Positive
HAART	no	On HAART	No HAART (except pregnancy prophylaxis)

Abbreviations: AA, African Americans; NVS, Natural Virus Suppressors; M, Male; F, Female; HAART, Highly Active Antiretroviral Therapy.

The populations were mostly African Americans (AA), except for a small percentage of progressors and controls, which was composed of Caucasians and Asians. HIV progressors contained a higher proportion of males and this reflects our HIV clinic populations.

**Table 2:** Genotypic and allelic frequencies of SNP rs1801274 (FCGR2A) and rs396991 (FCGR3A) in HIV negative controls, NVS and HIV Progressors.

	Controls	HIV positives	
		NVS	Progressors
FCGR2A			
HH	4(13%)	9(20%)	4(11%)
HR	21(65%)	26(57%)	20(56%)
RR	7(22%)	10(22%)	12(33%)
FCGR3A			
FF	36(51%)	14(33%)	21(36%)
FV	32(46%)	29(67%)	32(54%)
VV	2(2.8%)	0(0%)	6(10%)

Abbreviations: SNP, Single Nucleotide Polymorphism; NVS, Natural Virus Suppressors.

FCGR genotypic variants (FCGR2A H131R and FCGR3A F158V) were studied in HIV progressors, NVS as well as HIV negative controls. Genotypic and allelic frequencies of rs396991 and rs1801274 are shown. The genotypic frequencies of the HIV sero-negative patients are in Hardy-Weinberg equillibrium.

**Table 3:** Association of SNPs rs1801274 (FCGR2A) and rs396991 (FCGR3A) with HIV status.

SNP	All HIV+# vs Controls			Progressors vs NVS		
	Genotype	p-value	OR (95% CI)	Genotype	p-value	OR (95% CI)
rs396991	V allele	0.0398	2.0 (1.07–3.73)	VV	0.038	ND*
rs396991+rs1801274	FF+RR	NS**		FF+RR	0.025	8.76(1.08–70.77)

Significant differences related to HIV status are shown. For FCGR3A, carriage of V allele was associated with increased risk of HIV seropositivity. Among HIV sero-positive cases, there was a significant association between VV genotype (i.e., lack of allele F) and the progressor phenotype. For the FCGR2A∶FCGR3A extended haplotype, the RR∶FF genotype was associated with disease progression.

Fisher's exact test (2-tailed) p values and Odds ratios are listed where significant.

*None of the NVS donors carried the F allele, so odds ratio were not calculated.

#Include Progressors and NVS.

**Not significant.

We used the largest sample set available to us for each group. This resulted in different numbers of samples for control, HIV progressors and elite controllers. Each volunteer was also asked to report racial or ethnic identity among several choices; these racial distributions were similar but not identical in each group. Both males and females were represented in all groups, although the females formed smaller percentage of the HIV progressor group, a reflection of our HIV clinic population.

Informed written consent was obtained from all patients. The Institutional Review Board of the University of Maryland approved the study.

### Genotyping methods

Genomic DNA from each individual was genotyped for SNP rs396991 and rs1801274 (in FCGR3A and FCGR2A, respectively) with the ABI prism snapshot reaction kit (Applied Biosystems, Foster City, CA) using PCR amplified gene products. For FCGR3A, gene-specific forward primer 5′-AGTTCATCATAATTCTGACTCCT-3′ and reverse primer 5′-ACCTTGAGTGATGGTGATGTTCA-3′ were used. The forward primer differed from FCGR3B by a single nucleotide at the 3′ end to provide gene specificity. For FCGR2A, the forward primer 5′GGAAAATCCCAGAAATTCTCCC-3′ and reverse primer 5′-CAACAGCCTGACTACCTATTACCTGG-3′ were used. The snapshot reaction was performed as per the instructions in the kit. The products were run on ABI Prism 3130 DNA sequencer and data were analyzed using the GeneMapper Analysis software (Applied Biosystems).

Results for representative samples were confirmed by direct sequencing of the PCR products using Big Dye Terminator V3.1 Cycle sequencing kit and a 3130 Genetic Analyzer (Applied Biosystems, Foster City, CA).
